# Case Report: The First Case of COVID-19 in Bhutan

**DOI:** 10.4269/ajtmh.20-0259

**Published:** 2020-04-20

**Authors:** Shankar LeVine, Guru Prasad Dhakal, Tshering Penjor, Pem Chuki, Kesang Namgyal, Tshokey, Melanie Watts

**Affiliations:** Jigme Dorji Wangchuck National Referral Hospital (JDWNRH), Khesar Gyalpo University of Medical Sciences of Bhutan (KGUMSB), Thimphu, Bhutan

## Abstract

The initial cases of novel coronavirus disease-19 (COVID-19) in a country are of utmost importance given their impact on healthcare providers, the country’s preparedness response, and the initial molding of the public perception toward this pandemic. In Bhutan, the index case was a 76-year-old immunocompromised man who had traveled from the United States and entered Bhutan as a tourist. He presented initially with vague gastrointerestinal symptoms and later a cough. His atypical presentation led to a delay in diagnosis, but ultimately he was isolated and tested. On confirming the diagnosis of COVID-19, the patient was isolated in a separate hospital with a dedicated medical care team. All contacts were traced and quarantined. The patient’s respiratory status deteriorated despite broad-spectrum antivirals, antibiotics, and intensive supportive care. He required intubation and was given a trial of intravenous immunoglobulin to modulate his likely aberrant immune response. Subsequently, the patient’s clinical status improved, and after 8 days of hospitalization, he was transferred out of the country, where he recovered. This was a learning experience for the treating medical staff, the government, and the people of Bhutan.

## INTRODUCTION

As novel coronavirus disease (COVID-19) rapidly spreads across the globe; the initial cases in a country and their outcomes may have a tremendous impact. In a small, relatively isolated country such as Bhutan, this impact may be felt not only by the patients and their families but also by the country’s population, as the case and the country’s response are keenly followed. This report describes the first patient diagnosed and treated for COVID-19 in Bhutan.

## CASE PRESENTATION

The index case was a 76-year-old American man, with a history of hypertension, hyperlipidemia, and neuropathy for which he was on medications; surgical history was notable for a splenectomy due to mantle cell lymphoma. The patient traveled from the United States through airports in London, Mumbai, and Kolkata, before arriving in Jorhat, Assam, India. Halfway through a week-long cruise on the Brahmaputra River in Assam, he started feeling ill. Following the cruise, he flew to Bhutan, and over the first 2 days in country, he sought medical care for intermittent symptoms including bloating, loss of appetite, diarrhea, and fatigue. On his fourth day in Bhutan, he presented to the emergency department in Thimphu, where he was noted to be afebrile but hypoxic, with an oxygen saturation of 78% on room air, and he endorsed 4 days of cough. Based on preparedness screening protocols, the patient was clinically screened for COVID-19, and the decision was made to isolate and test the patient. Initial chest X-ray revealed mild bilateral patchy infiltrates, and he was started on oseltamivir, ceftriaxone, and doxycycline, and was kept in respiratory isolation on supplemental oxygen.

The patient’s swab reverse transcriptase–polymerase chain reaction test for COVID-19 was confirmed positive at midnight, and by morning, the Ministry of Health had traced approximately 90 contacts from the healthcare sector and hotel and restaurant staff; based on their degree of exposure, the patient contacts were instructed to either home quarantine or present to a quarantine facility. On royal command, a recently constructed ophthalmology hospital building was rapidly converted into a COVID-19 treatment facility including separate equipment, medications, and staffing. A dedicated care team was assembled comprising an emergency critical care physician, two internal medicine physicians, and nurses.

**Figure 1.**
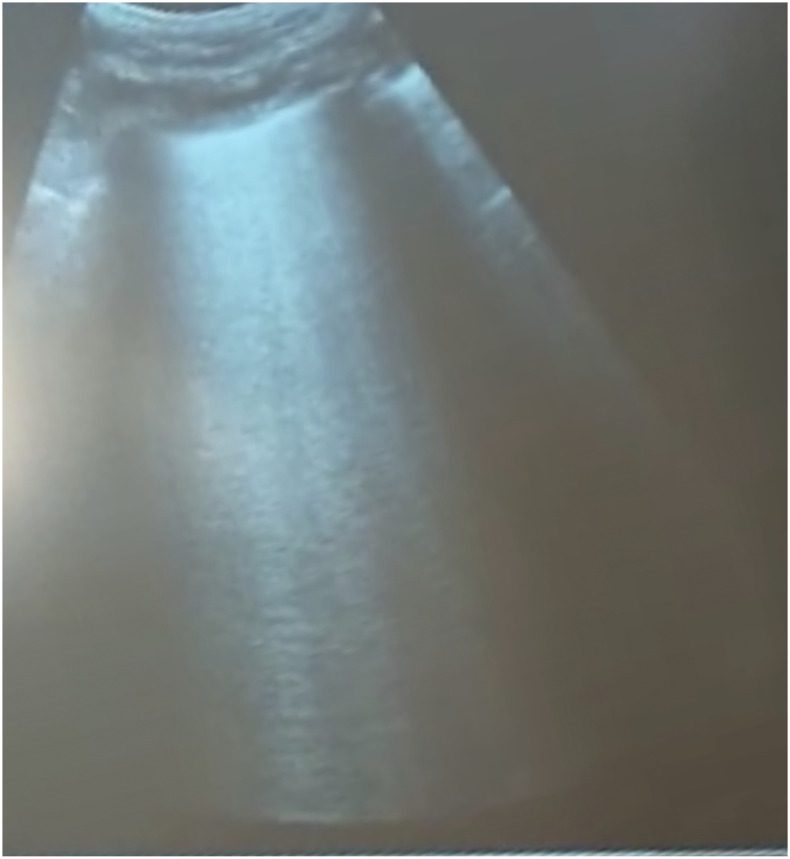
B lines were followed on point-of-care ultrasound documenting the worsening acute respiratory distress syndrome.

**Figure 2.**
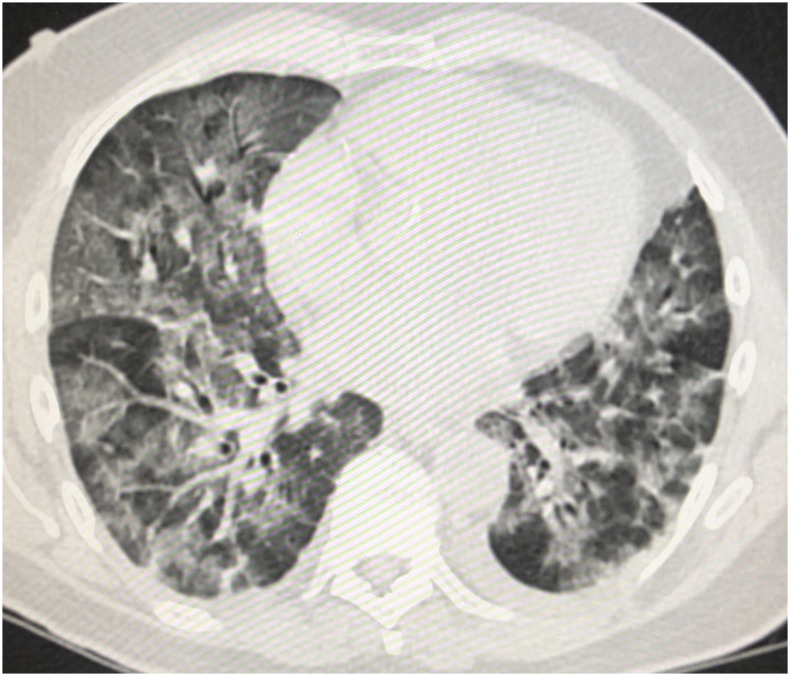
Computed tomography revealing bilateral infiltrates consistent with acute respiratory distress syndrome.

Over the first days of hospitalization, the patient’s oxygen requirement gradually worsened; his white blood cell (WBC) count and C-reactive protein (CRP) continued to increase. Point-of-care ultrasound revealed worsening B lines (Figure 1). Despite the addition of lopinavir and ritonavir and switching antibiotics to meropenem and vancomycin, he developed and maintained a low-grade fever. Computed tomography of the chest revealed diffuse ground-glass opacities consistent with acute respiratory distress syndrome (ARDS) (Figure 2). On the fifth day after diagnosis, the patient’s oxygen requirement and work of breathing dramatically worsened, and he was intubated and placed on a ventilator for respiratory support.

**Figure 3.**
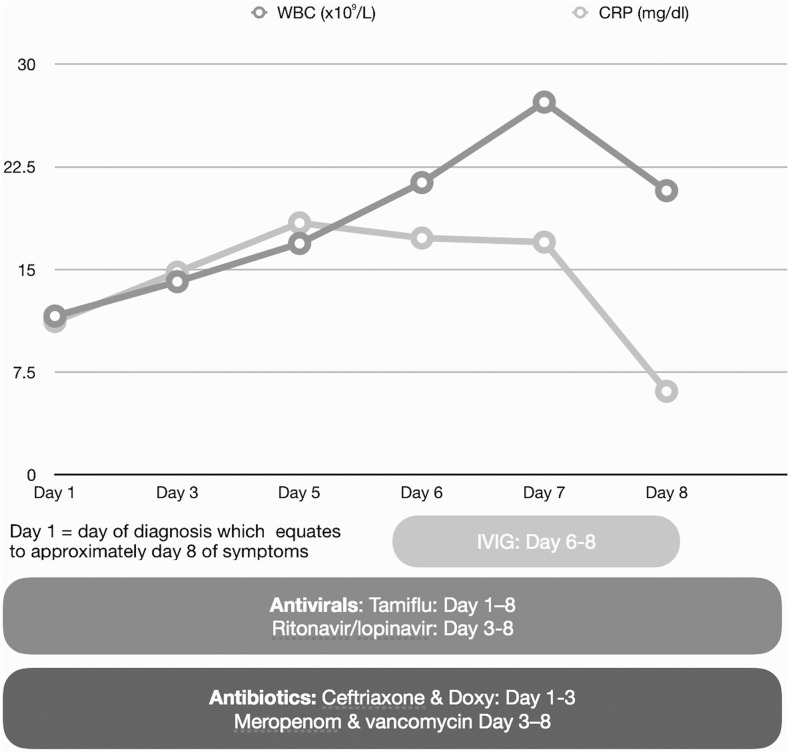
Graph demonstrating white blood cell and C-reactive protein in relation to days of illness and timing of treatments given.

Having reviewed the data of intravenous immunoglobulin (IVIG) in the setting of ARDS,^1,2^ a decision was made to give a 3-day course of 0.5 g/kg (40 g daily). The dose was largely based on the limited quantity of available IVIG. The patient received his first dose of IVIG on the sixth day after diagnosis and underwent prone positioning for ARDS.^3^ By the next morning, his oxygen requirement had improved, and 48 hours after the first dose of IVIG, the patient’s WBC count and CRP had decreased (Figure 3).

After 8 days of treatment and significant clinical improvement in Bhutan, the patient was evacuated to his home country. His clinical status continued to improve, and he was extubated 7 days after evacuation. Fifteen days after his initial positive COVID-19 test and hospitalization, he no longer required oxygen therapy and rehabilitative therapy was initiated.

## DISCUSSION

As the COVID-19 pandemic continues to spread to new countries, some aspects pertaining to the patient’s presentation, interventions to provide patient care, and the country’s measures of containment in response to this first case in Bhutan may be of broad interest.

The patient’s initial presenting symptoms were gastrointestinal; he subsequently developed a cough. Although early studies suggested that gastrointestinal symptoms were uncommon with COVID-19, subsequent studies have suggested that such symptoms are more common, noted in 10% and 18.6% in two studies.^4–6^ The patient’s lack of appetite prompted him to seek medical care. In a recent study, 78.6% of COVID-19 patients suffered from a lack of appetite at presentation.^6^ The patient’s presentation did not fall into the case definition for COVID-19 that Bhutan was using at that time, which was limited to fever and respiratory symptoms. This case is a reminder of atypical presentations and the need for allowing medical providers’ clinical discretion in management and for regular updating of case definitions during a new disease outbreak.

As the patient’s clinical status worsened, it seemed possible that this may have been due to an aberrant immune response, which is described in patients with viral infections, leading to ARDS.^2,7^ Prior case reports of IVIG successfully treating ARDS secondary to viral illnesses have been documented.^1,2^ After receiving IVIG, the patient had dramatic improvement of oxygenation and a downward trend in his inflammatory markers. Although the efficacy of IVIG, timing, dosage, and patient selection all clearly require further study, this case suggests that a trial of IVIG for the treatment of COVID-19 is warranted.

The public health strategies initiated by Bhutan’s Ministry of Health and government are beyond the scope of this case report, but the immediate steps pertaining to the patient’s contacts and medical staff caring for the patient are of interest. Some relevant lessons from Singapore and Hong Kong, which were able to curtail the rapid spread of the virus fairly effectively, include the need for the following: 1) appropriate personal protective equipment for medical staff in contact with a suspected patient, 2) disinfecting surfaces, 3) treating patients in a separate ward or hospital with a separate team, and 4) contact tracing and quarantine.^8,9^ In Singapore, contact tracing accounted for the primary detection of approximately half of the country’s first 100 COVID-19 patients.^8^

Despite the patient’s atypical presentation, Bhutan’s preparedness played a role in limiting exposure and making the initial diagnosis. The triage nurse, trained to screen patients for recent travel, alerted the emergency physician, who then took the patient to a predesignated isolation area for suspected cases. After the patient tested positive, a decision was rapidly made to designate a separate hospital building as a dedicated COVID-19 hospital. Separate staff including doctors, nurses, ambulance drivers, and cleaners were designated for this facility. These staff lived in a separate facility for the duration of treating the patient, after which they were put in a 14-day quarantine and monitored for symptoms. All of the patient’s contacts were traced and quarantined. Those with exposures deemed high risk were tested initially and at the end of their 14 days of quarantine. Although this is not common practice globally, it led to the diagnosis of COVID-19 infection in the patient’s partner, who was asymptomatic. No other initial contacts or medical staff tested positive by the end of their 14-day quarantine.

To prevent the import of cases and subsequent local transmission from occurring, Bhutan rapidly initiated travel restrictions barring the entry of all nonnationals and instituted a mandatory quarantine in designated hotels for all persons entering Bhutan. To date, there have been five cases of COVID-19; the three additional cases were diagnosed among repatriated quarantined Bhutanese students who were studying abroad. There has been no evidence of local community spread.
